# DEVELOPING OF AN SDM NURSING PRACTICE MODEL FOR OLDER PATIENTS WITH DEMENTIA IN ACUTE CARE HOSPITALS

**DOI:** 10.1093/geroni/igae098.2914

**Published:** 2024-12-31

**Authors:** Taeko Saito, Harue Masaki

**Affiliations:** Nippon Medical School, Inzai, Chiba, Japan; Chiba University, Chiba, Chiba, Japan

## Abstract

This study aims to implement a Shared Decision-Making (SDM)nursing practice model for older patients with dementia in acute care hospitals among acute care nurses and evaluate its feasibility. As a preliminary step, a hybrid concept analysis was conducted, and a SDM nursing practice model was developed and refined based on the results of semi-structured interviews with nursing practitioners and researchers. In this study, five acute care nurses were recruited as research participants to confirm the feasibility of this model. Data analysis involved conducting content analysis from verbatim transcripts of individual interviews with the five participants after practice to assess feasibility. Additionally, both before and after the practice, surveys based on SDM-Q9 and Group’s SDM, validated tools for assessing the SDM process, were administered to the five research participant nurses and five health care professionals involved in support (MD, MSW), generating data from the perspectives of users and stakeholders. The results indicated the feasibility of implementing the SDM process within the constraints of the acute care setting. Moreover, the study demonstrated that the SDM nursing practice model effectively adheres to the SDM process during acute phases, thereby clarifying the role of clinical nurses in facilitating the Sharad decision-making for older patients with dementia.

